# Friedreich’s ataxia-associated childhood hypertrophic cardiomyopathy: a national cohort study

**DOI:** 10.1136/archdischild-2021-322455

**Published:** 2021-10-05

**Authors:** Gabrielle Norrish, Thomas Rance, Elena Montanes, Ella Field, Elspeth Brown, Vinay Bhole, Graham Stuart, Orhan Uzun, Karen A McLeod, Maria Ilina, Satish Adwani, Piers Daubeney, Grazia Delle Donne, Katie Linter, Caroline B Jones, Tara Bharucha, Elena Cervi, Juan Pablo Kaski

**Affiliations:** 1 Centre for Inherited Cardiovascular Disease, Great Ormond Street Hospital For Children NHS Foundation Trust, London, UK; 2 Institute of Cardiovascular Science, University College London, London, UK; 3 Paediatric Cardiology, Leeds General Infirmary, Leeds, UK; 4 Paediatric Cardiology, Birmingham Women and Children’s NHS Foundation Trust, Birmingham, UK; 5 Bristol Congenital Heart Centre, Bristol Heart Institute, Bristol, UK; 6 Paediatric cardiology, University Hospital of Wales, Cardiff, UK; 7 Paediatric cardiology, Royal Hospital for Sick Children, Glasgow, UK; 8 Paediatric cardiology, Royal Hospital for Children, Glasgow, UK; 9 Paediatric Cardiology, John Radcliffe Hospital, Oxford, UK; 10 Paediatric cardiology, Royal Brompton and Harefield NHS Trust and National Heart and Lung Institute, London, UK; 11 Paediatric cardiology, University Hospitals of Leicester NHS Trust, Leicester, UK; 12 Paediatric cardiology, Alder Hey Children’s Hospital, Liverpool, UK; 13 Department of Congenital Cardiology, University Hospital Southampton NHS Foundation Trust, Southampton, UK

**Keywords:** cardiology, paediatrics, neurology

## Abstract

**Objective:**

Hypertrophic cardiomyopathy (HCM) is an important predictor of long-term outcomes in Friedreich’s ataxia (FA), but the clinical spectrum and survival in childhood is poorly described. This study aimed to describe the clinical characteristics of children with FA-HCM.

**Design and setting:**

Retrospective, longitudinal cohort study of children with FA-HCM from the UK.

**Patients:**

78 children (<18 years) with FA-HCM diagnosed over four decades.

**Intervention:**

Anonymised retrospective demographic and clinical data were collected from baseline evaluation and follow-up.

**Main outcome measures:**

The primary study end-point was all-cause mortality (sudden cardiac death, atrial arrhythmia-related death, heart failure-related death, non-cardiac death) or cardiac transplantation.

**Results:**

The mean age at diagnosis of FA-HCM was 10.9 (±3.1) years. Diagnosis was within 1 year of cardiac referral in 34 (65.0%) patients, but preceded the diagnosis of FA in 4 (5.3%). At baseline, 65 (90.3%) had concentric left ventricular hypertrophy and 6 (12.5%) had systolic impairment. Over a median follow-up of 5.1 years (IQR 2.4–7.3), 8 (10.5%) had documented supraventricular arrhythmias and 8 (10.5%) died (atrial arrhythmia-related n=2; heart failure-related n=1; non-cardiac n=2; or unknown cause n=3), but there were no sudden cardiac deaths. Freedom from death or transplantation at 10 years was 80.8% (95% CI 62.5 to 90.8).

**Conclusions:**

This is the largest cohort of childhood FA-HCM reported to date and describes a high prevalence of atrial arrhythmias and impaired systolic function in childhood, suggesting early progression to end-stage disease. Overall mortality is similar to that reported in non-syndromic childhood HCM, but no patients died suddenly.

## Abstract (precis)

In the largest cohort of children with Hypertrophic Cardiomyopathy associated with Friedrich's Ataxia presented to date a surprisingly high prevalence of atrial arrhythmias are described, and a reminder of the need for serial cardiac screening in these children is reinforced.

**What is already known on this topic?**

- Hypertrophic cardiomyopathy (HCM) is an important predictor of long-term outcomes in Friedreich’s ataxia (FA).
- HCM often presents many years after the diagnosis of ataxia but FA accounts for up to 10% of childhood HCM.
- The clinical spectrum and survival of HCM caused by FA childhood is poorly described.

**What this study adds?**

- In this national cohort, age of presentation and phenotype were variable, but included a high prevalence of atrial arrhythmias and early progression to end-stage disease.
- Overall mortality was similar to that reported in non-syndromic childhood HCM, but no patients died suddenly.
- Routine, serial cardiac screening should be performed for all children with a diagnosis of FA as the cardiac prognosis is not benign during childhood.

## Introduction

Hypertrophic cardiomyopathy (HCM) is characterised by left ventricular (LV) hypertrophy in the absence of abnormal loading conditions sufficient to explain the abnormality.^1^

Friedreich’s ataxia (FA) is a progressive neurodegenerative movement disorder characterised by cerebellar ataxia, dysarthria, areflexia and muscle weakness.^2^ ^3^ HCM occurs in up to 85% of patients with FA and cardiac involvement has been shown to be an important predictor of long-term outcomes.^4–6^ Although HCM usually presents many years after the diagnosis of ataxia, small childhood series have reported HCM in up to two-thirds of patients, and FA accounts for up to 10% of cases of childhood HCM.^7–10^ However, the clinical spectrum of disease and survival in childhood onset FA-HCM is poorly described, and our understanding of disease progression is limited. The aim of this study was to describe the clinical characteristics of children with FA-HCM over four decades in a well-characterised UK cohort.

## Methods

A retrospective, longitudinal multicentre cohort of children with FA-HCM diagnosed under the age of 18 years was identified from a previously published national paediatric HCM cohort from the UK.^8^

### Clinical evaluation and data collection

Anonymised, non-invasive clinical data were collected from baseline cardiac evaluation and during follow-up: demographics, age at diagnosis (FA and HCM), family history, cardiac symptoms, medical therapy, 12-lead and ambulatory ECG, and two-dimensional, Doppler and colour echocardiography. The diagnosis of FA was made in all cases following clinical assessment and investigation by a paediatric neurologist. The diagnosis of HCM was accepted if the maximal left ventricular wall thickness (MLVWT) in any myocardial segment was greater than 2 SD above the body surface area (BSA)-corrected population mean (z-score ≥+2)^1^ (see online supplemental methods).

10.1136/archdischild-2021-322455.supp1 Supplementary data

### Outcomes

The primary study end-point taken from the last clinic appointment was all-cause mortality (sudden cardiac death (SCD), atrial arrhythmia-related death (acute haemodynamic decompensation in the presence of an atrial tachycardia), heart failure-related death, non-cardiac death) or cardiac transplantation. The secondary outcomes were presence of arrhythmias detected on ambulatory or inpatient ECG monitoring (atrial or ventricular).

### Statistical analysis

BSA was calculated from height and weight.^11^ MLVWT measurements are expressed in millimetres and z-scores relative to the distribution of measurements for BSA in healthy children.^12^ Normally distributed continuous variables are described as mean±SD and comparisons were made using Student’s t-test or Wilcoxon rank-sum. Skewed data are described as median (IQR) and comparisons were made using χ^2^ test or Fisher’s exact test as appropriate. Estimates for transplant-free survival and survival free from arrhythmias were calculated using the Kaplan-Meier product limit method. The association of clinical variables with the outcome of interest was assessed in a univariate proportional hazards model. All statistical analyses were performed using STATA V.14.

## Results

**Figure 1:**
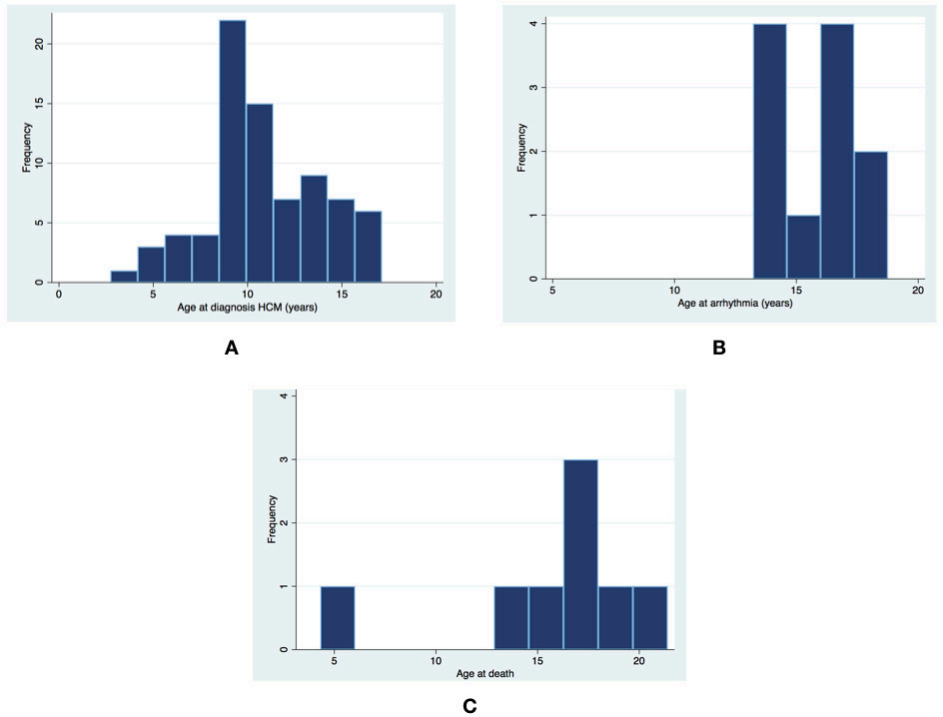
Age at the time of (A) hypertrophic cardiomyopathy (HCM) diagnosis, (B) ventricular or atrial arrhythmia, and (C) death.

Seventy-eight patients with FA-associated HCM were identified (male n=42, 53.9%), with a mean age at diagnosis of FA and HCM of 9.98±3.08 (n=62) and 10.87±3.08 (n=78), respectively. The diagnosis of HCM was made under the age of 10 years in 34 (43.6%) patients (figure 1). In four patients (5.1%), the cardiac diagnosis preceded the diagnosis of FA by a median of 2.9 years (range 5.1–0.2 years) following referral for cardiac symptoms (chest pain n=1, palpitations and murmur n=1, or heart failure n=2). Of the remaining patients, 34 (65.0%) met the diagnostic criteria for HCM within 1 year of cardiac referral for confirmed or suspected FA. Data on genetic testing were available in 45 patients (homozygous triplet expansion n=41, heterozygote n=1, unknown n=3). Two patients had not undergone genetic testing, and genetic testing status data were not available for the remaining 31 patients. Information on the length of triplet expansion was not available.

### Baseline clinical assessment

At the time of baseline cardiac assessment, 74 (94.9%) patients met the diagnostic criteria for HCM and 48 (66.7%) were asymptomatic. Of those with symptoms, 11 (39.3%) had chest pain, 6 (21.4%) had palpitations, 6 (21.4%) had presyncope or syncope, and 11 (39.3%) had heart failure symptoms. Twelve patients (16.4%) were started on cardiac medications (beta-blockers n=8, diuretics n=2, ACE inhibitors n=1, digoxin n=1) and 12 (16.4%) were receiving antioxidants (idebenone n=9, coenzyme Q10 n=3). The echocardiographic and ECG characteristics at baseline are described in table 1. Of those with available echocardiographic assessment of diastolic function (n=39), heart failure symptoms were not more common in those with abnormal diastolic parameters (n=1 (25%) vs 5 (14.3%), p=0.574) (online supplemental table 1). Twelve-lead resting ECG was available for 41 patients (52.5%); only 2 patients (4.9%) had no ECG abnormalities at baseline. Patients presenting in more recent era (2010 onwards) were older but did not otherwise differ in baseline characteristics (online supplemental table 2).

**Table 1:** Clinical phenotype at baseline and follow-up

Echocardiographic characteristics		Baseline, n=76	Follow-up, n=64
LV hypertrophy	Mean MLVWT, mm (±SD, range) (n=71)	12.8 (±2.6, 8.0–19.0)	13.0 (±3.0, 7.0–24.0)
	Mean MLVWT z-score (±SD) (n=34)	6.6 (±3.4)	NA*
Pattern of hypertrophy (n=72)	Concentric, n (%)	65 (90.3)	58 (90.6)
	ASH, n (%)	7 (9.7)	5 (7.8)
	Eccentric, n (%)	0	1 (1.6)
Median maximal LVOT gradient (mm Hg) (IQR) (n=50)/(n=45)		6 (4–9)	6 (4–9)
LV end diastolic dimension	Mean LVEDd, mm (±SD, range) (n=56)	36.0 (±6.2, 25–54)	37.9 (±6.3)
	Median LVEDd z-score (IQR)	−1.5 (−2.5 to −0.5)	NA*
Impaired LV systolic function (n=49), n (%)		6 (12.5)	3 (6.3)
Impaired LV diastolic function (n=39), n (%)		6 (15.4)	6 (18.2)
**ECG characteristics**		**Baseline, n=76**	**Follow-up, n=29**
Axis	Normal, n (%)	33 (82.5)	19 (70.4)
	Right, n (%)	6 (15.0)	7 (29.2)
	Left, n (%)	0	0
	Extreme, n (%)	1 (2.5)	1 (3.4)
PR interval	Mean (ms)	136 (±24.3)	137.5 (±25.7)
	Range	104–200	93–200
Sokolow-Lyon score	Mean±SD (range), mm	38.5±13.9 (14–67)	31.5±14.5 (9–70)
	≥35 mm, n (%)	25 (64.1)	10 (34.5)
Dominant S wave in V4	n (%)	11 (27.5)	7 (24.1)
QT interval	Mean (±SD)	316.1 (46.3)	322.3 (50.0)
Corrected QT interval	Mean Qtc (±)	375.7 (46.1)	370.1 (40.3)
	≥440 ms, n (%)	3 (7.3)	2 (6.9)
T wave inversion	Present, n (%)	30 (73.2)	25 (86.2)
Location of T wave inversion	Inferior	4	3
	Lateral	2	0
	Inferolateral	20	18
	Anterior	3	1
	Anterolateral and inferior	1	3
ST segment changes	Elevation, n (%)	5 (12.2)	6 (20.7)
	Depression, n (%)	2 (4.9)	1 (3.4)
Pathological Q waves	Present, n (%)	11 (26.8)	13 (44.8)
Location of pathological Q waves	Inferolateral	5	1
	Inferior	6	12

ASH = asymmetric septal hypertrophy, LVEDd = Left ventricular end diastolic dimension

*The z-scores for MLVWT were not available for follow-up echocardiograms (missing weight and height data).

LV, left ventricular; LVOT, left ventricular outflow tract; MLVWT, maximal left ventricular wall thickness; NA, not available.

### Atypical presentation of FA-HCM

**Figure 2:**
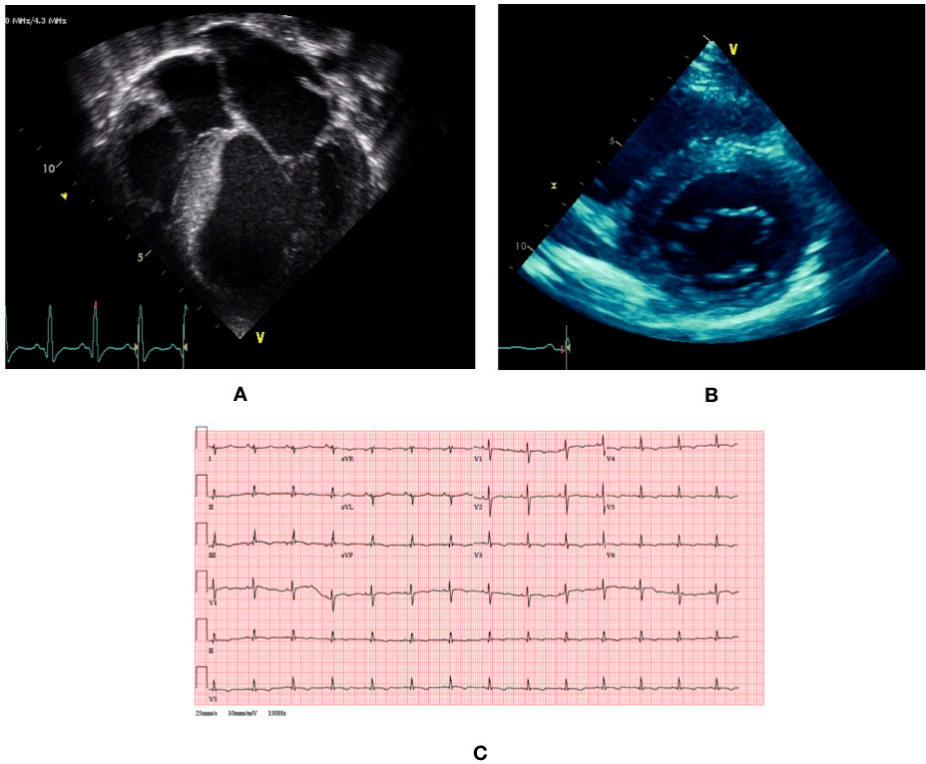
Clinical phenotype of a patient with atypical presentation of Friedreich’s ataxia-associated hypertrophic cardiomyopathy. (A) Transthoracic echocardiogram at presentation at age 5 shows dilated and hypertrophied phenotype with impaired systolic function. (B) Transthoracic echocardiogram at age 15 shows concentric hypertrophy with maximal left ventricular wall thickness of 14 mm. (C) 12-lead ECG at age 15 shows small voltages, right axis deviation and widespread repolarisation abnormalities (flat or inverted T waves inferiorly and V2–V6).

Two patients had an atypical presentation under the age of 5 years with a dilated hypokinetic cardiomyopathy phenotype, both of whom were diagnosed with FA, with genetic confirmation, several years later. The first of these patients presented with a presumed viral myocarditis and had persistent LV systolic dysfunction, for which they underwent heart transplantation. Extensive investigation (including viral serology and metabolic screen, including muscle biopsy) was performed at presentation to identify the underlying aetiology at the time of presentation. The results of cardiac biopsy from the time of cardiac transplantation were not available for analysis. The patient subsequently developed ataxia and was diagnosed with FA 3 years after the diagnosis of HCM. The second patient presented with symptoms of heart failure and a dilated and hypertrophied heart on echocardiography, with impaired systolic function (fractional shortening 8%). She was initially listed for heart transplantation but systolic function recovered with medical therapy and she was removed from the transplant list. A diagnosis of FA was made 5 years after presentation. The cardiac phenotype initially stabilised, although she remained symptomatic (chest pain and palpitations) and had ventricular arrhythmias (non-sustained ventricular tachycardia (NSVT)) detected on ambulatory ECG monitoring. During adolescence she has had progressive reduction in LV systolic function (ejection fraction 53%) and LV wall thinning (figure 2).

### Follow-up

The median length of follow-up was 5.1 years (IQR 2.4–7.3). The median age at last clinical follow-up was 16.3 years (IQR 14.25–18.35); 19 (24.4%) patients had been transitioned to adult services. A phenotype of HCM developed in the four patients with no hypertrophy at baseline over a median of 2.6 years (range 1.3–4.8).

### Follow-up clinical assessment

At last clinical assessment (n=76), 33 (54.5%) were symptomatic. Forty patients (56.3%) were taking cardiac medications and 23 (32.4%) were taking antioxidant medication. Echocardiographic and ECG characteristics are described in table 1. Mean MLVWT did not differ significantly from baseline assessment (p=0.255). Of the five patients with LV systolic impairment at baseline with repeat assessment, LV function normalised in three, remained mildly impaired in one (ejection fraction 53%) and deteriorated necessitating cardiac transplantation at age 4 in one.

### Outcomes

#### Arrhythmic events

One or more ambulatory or inpatient ECG recordings were available for 51 (70.8%) patients, of whom 39 (76.5%) had no arrhythmias detected. Eight (15.7%) had atrial arrhythmias at a median age of 16.3 (IQR 13.4–17.8) (figure 1B): atrial fibrillation (n=3), atrial flutter (n=1), atrial ectopic tachycardia (n=1) and unspecified supraventricular tachycardia (n=3). Two developed decompensated heart failure secondary to atrial arrhythmias and died. Atrial arrhythmias occurred at an annual rate of 1.85% (95% CI 0.93 to 3.70). There were no differences in baseline clinical characteristics between patients with and without atrial arrhythmias, but a higher proportion had impaired systolic function at last clinical review (n=3 (37.5%) vs n=1 (1.4%), p<0.001) (online supplemental table 3). No demographic or baseline clinical characteristics predicted survival free from atrial arrhythmias on univariable analysis (table 2). NSVT was detected in four patients (7.8%). No patients underwent implantation of an implantable cardioverter defibrillator device and there were no SCDs. The clinical phenotype and outcomes of patients with atrial or ventricular arrhythmias are described in table 3.

**Table 2:** Univariate Cox regression analysis for predictors of outcomes

Clinical predictor	Mortality or cardiac transplantation		Atrial arrhythmia	
	HR (95% CI)	P value	HR (95% CI)	P value
Male gender	0.495 (0.11 to 2.21)	0.357	0.483 (0.11 to 2.11)	0.324
Any symptoms at baseline	1.053 (0.19 to 5.79)	0.953	0.904 (0.16 to 4.95)	0.907
Heart failure symptoms	0.888 (0.11 to 7.41)	0.912	0.862 (0.11 to 7.02)	0.887
Increasing LVMWT	0.735 (0.49 to 1.10)	0.089	0.946 (0.72 to 1.24)	0.687
Increasing LVOT gradient	0.956 (0.80 to 1.14)	0.516	0.936 (0.75 to 1.17)	0.418
Impaired LV systolic function	3.162 (0.52 to 19.11)	0.237	1.360 (0.15 to 12.20)	0.790
Impaired LV diastolic function	NA	NA	1.620 (0.17 to 15.75)	0.690
Atrial arrhythmia	1.834 (0.37 to 9.12)	0.482	NA	NA

LV, left ventricular; LVMWT, left ventricular maximal wall thickness; LVOT, left ventricular outflow tract; NA, not available.

**Table 3:** Clinical phenotype and outcomes of patients with atrial and ventricular arrhythmias

Age at diagnosis of HCM	Arrhythmia	Age at first arrhythmia	Cardiac phenotype at presentation	Clinical information	Outcome
9	SVT and AF	14	Concentric LVH. No LVOTO. No systolic impairment.	Palpitations with SVT on ambulatory ECG. Treated with bisoprolol and diltiazem. At age 18 AF requiring DC cardioversion and therapy change (amiodarone).	Alive.
15	AF	18	Concentric LVH. No LVOTO. MWT 10 mm.	Fast AF with decompensated heart failure. Treated with diuretics, amiodarone and digoxin increased. At age 19 fast AF requiring DC cardioversion.	Died secondary to heart failure at age 19.
10	AET	11	Concentric LVH. No LVOTO.	Palpitations with AET on ambulatory ECG. Treated with verapamil.	Alive.
10	SVT	13	Concentric LVH. No systolic impairment.	Asymptomatic. Ambulatory monitoring showed SVT. Treated with B-blockers.	Alive.
7	Atrial flutter	28	Concentric LVH. MWT 14 mm.	Unknown clinical presentation.	Transitioned to adult care.
10	AF	19	Concentric LVH. MWT 12. No LVOTO. No systolic impairment.	Presented with chest pain and palpitations.	Transitioned to adult care.
11	Atrial flutter	13	Echo showed reduced EF and FS. Concentric LVH. No LVOTO. MWT 11 mm.	Palpitations with atrial flutter in the context of viral illness. Treated with digoxin and DC cardioversion. Maintenance therapy of flecainide, lisinopril and aspirin.	Transitioned to adult care.
10	AF with fast ventricular conduction	17	Mild concentric LVH. No LVOTO. MWT 13 mm.	Fast AF postoperatively with lactic acidosis.	Died secondary to decompensated heart failure at age 17.
5	NSVT	14	Presented at age 5 with symptoms suggestive of dilated cardiomyopathy, listed for transplant. Concentric LVH. MWT 12 mm. Systolic impairment. No LVOTO.	Asymptomatic. Treated with amiodarone.	Alive.
14	NSVT	16	Concentric LVH. MWT 11 mm. No LVOTO. No systolic impairment.	Detected during episode of pancreatitis.	Alive.
11	NSVT	12	Concentric LVH. No systolic impairment. No LVOTO. MWT 15 mm.	Palpitations with NSVT on ambulatory ECG. Treated with amiodarone.	Died at age 32, unknown cause.
9	NSVT	17	Concentric LVH. MWT 15 mm.	Asymptomatic. Treated with nifedipine.	Alive.

AET, atrial ectopic tachycardia; AF, atrial fibrillation; DC, direct current; EF, ejection fraction; FS, fractional shortening; HCM, hypertrophic cardiomyopathy; LVH, left ventricular hypertrophy; LVOTO, left ventricular outflow tract obstruction; MWT, maximal wall thickness; NSVT, non-sustained ventricular tachycardia; SVT, supraventricular tachycardia.

#### Mortality and cardiac transplantation

Seventy patients (89.7%) were alive at last clinical review. Eight patients (10.6%) died (figure 1B): atrial arrhythmia-related (n=2), heart failure-related (n=1), non-cardiac (n=2) and unknown cause (n=3). Overall mortality or cardiac transplantation rate was 1.72 per 100 patient years (95% CI 0.86 to 3.44). Freedom from death or transplantation at 5 and 10 years was 96.5% (95% CI 86.4% to 99.1%) and 80.8% (95% CI 62.5 to 90.8), respectively. No demographic or baseline clinical characteristics predicted transplant-free survival on univariable analysis (table 2 and online supplemental figure 1).

10.1136/archdischild-2021-322455.supp2 Supplementary data

## Discussion

This study describes the clinical presentation, phenotype and outcomes of a large national cohort of patients with FA-HCM. Novel findings include a high prevalence of atrial arrhythmias and impaired systolic function in childhood, suggesting early progression to end-stage disease. Overall mortality was comparable with non-syndromic childhood HCM, but no sudden deaths occurred.

### Clinical presentation

Retrospective population-based studies have described FA-HCM to present in late childhood or early adulthood, in contrast to other syndromic causes of HCM, such as the RASopathies.^8–10^ The largest childhood series to date described the longitudinal course of 28 patients, not all of whom developed HCM during follow-up.^7^ The present manuscript describes a unique national cohort of patients with childhood FA-HCM and represents the largest reported study of this disease. In keeping with previous reports, the mean age of presentation was over the age of 10 and no patients presented in infancy. Nevertheless, two-fifths of patients presented in preadolescence (<10 years of age) and three (4%) presented under the age of 5 years. In addition, a small proportion of patients were diagnosed with HCM prior to the diagnosis of FA. These findings highlight the importance of including FA in the differential diagnosis for all childhood HCM, regardless of the age of presentation and presence of neurological features. Two-thirds were asymptomatic when first seen, which supports the practice of routine serial cardiac screening for all patients with FA.

### Phenotype of childhood FA-HCM

In agreement with previous retrospective studies,^5 7 10 13 14^ the predominant phenotype at baseline was that of concentric left ventricular hypertrophy (LVH) with no left ventricular outflow tract obstruction, and no patient had extreme LVH. No significant progression of hypertrophy was seen during follow-up, which supports previous reports of childhood being a time of phenotype stability in FA-HCM.^7 15^ However, a higher proportion of patients had impaired LV systolic function compared with non-syndromic childhood HCM populations,^9 10 16^ and two patients had an atypical presentation with a dilated hypokinetic cardiomyopathy phenotype. FA-HCM is known to progress to a hypokinetic dilated phase over follow-up^14 17^ and heart failure is one of the most common causes of mortality in adults,^6^ but this has not been previously described in childhood series.^7^ Our results support the hypothesis that childhood FA-HCM could be associated with a more severe phenotype, with early progression to, or more rarely presentation with, end-stage disease in childhood.^17 18^ LV function recovered in half of the patients with systolic impairment at baseline. This could be explained by the introduction of medical therapy, such as heart failure pharmacological treatments, but could also reflect a waxing and waning of disease severity as observed in other mitochondrial diseases.

### Outcomes

This study shows that overall mortality or cardiac transplantation rates in FA-HCM are similar to unselected childhood HCM populations,^8–10^ but the causes of death differed. Although FA is a multisystemic disease, cardiovascular involvement is an important contributor to long-term outcomes, and the most common causes of death reported in postmortem studies are heart failure and arrhythmic events.^6 19 20^ Cardiac mortality is described to occur at a younger mean age compared with non-cardiac deaths, but has rarely been reported to occur during childhood.^6 19^ In our data, although 25% of deaths were non-cardiac, 38% were secondary to cardiac (arrhythmia-related or heart failure), highlighting that the cardiac prognosis for this population is not benign in childhood and adolescence. No baseline clinical or demographic features were associated with mortality in this population in contrast to previous adult cohorts, which identified (left ventricular ejection fraction (LVEF) and LV mass as predictors of survival.^20^

Patients with FA-HCM are considered to be at low risk of malignant ventricular arrhythmias,^8 21 22^ and in keeping with this no patients in this cohort experienced sustained ventricular arrhythmias or died suddenly. This is in contrast to unselected childhood HCM populations where, outside of infancy, the most common cause of death is SCD.

A major novel finding in this study is the high prevalence of atrial arrhythmias in childhood FA-HCM, which were detected from adolescence onwards. No baseline clinical or demographic features were associated with arrhythmic events, but impaired systolic performance at the most recent clinical evaluation was more common in those with arrhythmias. This supports previous reports in small adult cohorts in which atrial arrhythmias were primarily seen in those with a hypokinetic dilated phenotype.^23^ Atrial arrhythmias, in particular atrial fibrillation, cause significant morbidity in adult-onset sarcomeric HCM but are rarely seen in childhood non-syndromic disease.^24^ This finding suggests that clinicians should regularly perform ambulatory ECG in all patients with FA-HCM, with particular focus on those with impaired or borderline LV systolic function.

### Limitations

This study is limited by problems inherent to all retrospective studies, in particular missing data. Patients were recruited from multiple centres over time, meaning that variations in patient assessment and management, including medical management of arrhythmias, are inevitable. This is also a strength of the study as it accurately reflects the historical and current outcomes of patients with childhood FA-HCM in the UK. It is beyond the scope of this study to describe the penetrance of FA-HCM in childhood as the cohort only included patients meeting the diagnosis for FA-HCM. Although this study describes a national cohort, the low incidence rate of FA-HCM means that the number of included patients was small, which reduced our power to detect statistically significant differences. Left atrial size is a recognised risk factor for developing atrial fibrillation in adults with HCM,^24^ although there are limited data in childhood-onset disease. Data on left atrial size were not systematically available in this patient cohort, preventing its exploration as a risk factor for atrial arrhythmias in this study. Previous studies have reported conflicting findings regarding the correlation between genotype and severity of cardiomyopathy.^7 13 20^ Genotype information was not available for all patients in this study, which prevented its investigation in this cohort. In addition, extended genetic testing was not performed in the two patients with atypical early presentation and the possibility of dual pathology cannot therefore be excluded.

## Conclusions

This national study is the largest multicentre description of FA-associated HCM during childhood and describes a symptomatic cohort with variable age of progression and cardiac phenotype. There was a high prevalence of atrial arrhythmias, most commonly in those with impaired LV systolic function, and early progression to end-stage disease. Overall mortality is similar to that reported in non-syndromic childhood HCM, but no patients died suddenly.

## Data Availability

The data underlying this article cannot be shared publically as consent for dissemination of patient data was not obtained. GN, TR and JPK had access to all data and final responsibility for submission of the manuscript. Data underlying this article is not available as consent was not obtained for sharing data.
