# Supplementary material for: Friedreich’s ataxia-associated childhood hypertrophic cardiomyopathy: a national cohort study
Source: Arch Dis Child. 2021 Oct 5;107(5):450–5. doi: 10.1136/archdischild-2021-322455 (PMC9046745; doi:10.1136/archdischild-2021-322455)
Supplement: Supplementary data [file archdischild-2021-322455supp001.pdf]

Supplementary methods

Echocardiographic measurements were made according to current guidelines (1). Specifically, MLVWT was measured in the parasternal long-axis or parasternal short-axis views (2D or M-Mode) at end diastole. Extreme left ventricular hypertrophy (LVH) was defined as a MLVWT ≥ 30mm or Z score ≥6 (1). LV diastolic dysfunction was assessed to be present if two out of four variables used to assess diastolic function (annular E' velocity, septal E' velocity, average E/E' ratio, LA diameter) were out of normal range for age and body surface area (2, 3). LV systolic dysfunction was defined as a LV fractional shortening ≤28% or ejection fraction ≤ 55% (4). Left ventricular outflow tract (LVOT) obstruction (LVOTO) was defined as a maximal LVOT gradient of ≥ 30mmHg (1).

12-lead ECGs were analysed by one observer (T.R) unaware of the clinical details of the patients. ECGs were excluded if trace quality was poor. Age-specific normal values for ECG parameters were used (5). The following parameters were measured (average of 3 beats) from lead II, or V5 if quality of trace was poor: heart rate (bpm), QRS axis, PR interval (ms), Sokolow-Lyon score (SV1 or SV2 + RV5 or RV6 ≥ 35mV)(6), QT interval (ms) and corrected QT interval (ms) using Bazett's formula(7) . The presence of the following parameters were described: dominant S wave in V4, pathological Q waves, pathological T wave inversion (>1mm beyond V1 aged ≥14 years, or beyond V3 aged < 14 years), ST segment depression (≥2mm in any lead), and ST segment elevation (≥2 mm in leads V1-V3, or ≥1mm in all other leads).

Non-sustained ventricular tachycardia (NSVT) during ambulatory ECG monitoring was defined as three or more consecutive ventricular beats at a rate of greater than 120 beats/min with a duration of less than 30 seconds(1).

Supplementary tables

Supplementary table 1: Clinical characteristics of patients with and without heart failure symptoms

		Heart failure symptoms (n=11)	No heart failure symptoms (n=61)	P value
**Baseline clinical assessment (n=72)**	**Any symptoms**	11 (100%)	13 (21.3%)	<0.001
	**Mean MLVWT (+/-SD)**	12.88 (2.03)	12.77 (2.65)	0.9105
	**Concentric hypertrophy**	9 (81.8%)	56 (91.8%)	0.304
	**LVOT obstruction (n=67)**	0 (0%)	2 (3.5%)	0.572
	**Systolic impairment (n=49)**	2 (33.3%)	5 (9.3%)	0.093
	**Diastolic impairment (n=39)**	1 (25.0%)	5 (14.3%)	0.574
		**Heart failure symptoms (n=11)**	**No heart failure symptoms (n=65)**	**P value**
**Last clinical assessment (n=76)**	**Any symptoms**	1 (12.5%)	10 (10.3%)	0.867
	**Mean MLVWT (+/-SD)**	12.82 (1.73)	13.01 (3.10)	0.883
	**Concentric hypertrophy (n=64)**	9 (90%)	59 (90.7%)	0.879
	**LVOT obstruction (n=17)**	1 (33.3%)	0 (0.0%)	0.026
	**Systolic impairment (n=47)**	1 (16.7%)	3 (7.3%)	0.443
	**Diastolic impairment (n=33)**	2 (50.0%)	8 (27.6%)	0.361

NYHA = New York Heart Association, LVOT = left ventricular outflow tract

Supplementary table 2: Baseline clinical characteristics of patients by era of presentation

		Pre-2000 (n=11)	2000 – 2009 (n=32)	2010 – 2018 (n=30)	P value
**Age of HCM diagnosis**		9.8 (+/-3.4)	10.6 (+/-2.2)	11.4 (+/-3.8)	0.013
**Baseline cardiac assessment**	Any cardiac symptoms	3 (42.9%)	10 (33.3%)	10 (33.3%)	0.882
	NYHA>2	2 (18.2%)	6 (18.8%)	2 (6.7%)	0.344
	LVMWT (mm)	12.9 (+/-2.2)	12.9 (+/-2.9)	12.3 (+/-2.8)	0.546
	LVMWT Z score	7.3 (+/-2.3)	7.2 (+/-4.3)	6.2 (+/-3.1)	0.393
	Systolic impairment	1 (16.7%)	4 (21.1%)	1 (4.2%)	0.23
**Atrial arrhythmia**		3 (27.3%)	5 (15.6%)	0 (0%)	0.025
**Mortality**		4 (36.4%)	4 (12.5%)	0 (0%)	0.004

Supplementary table 3: Clinical characteristics of patients with and without atrial arrhythmias

		Atrial arrhythmia (8)	No atrial arrhythmia (70)	P value
**Baseline clinical assessment (n=78)**	**Any symptoms**	2 (25%)	22 (31.4%)	0.153
	**NYHA >2**	1 (12.5%)	10 (14.3%)	0.891
	**Mean MLVWT (+/-SD)**	12.88 (2.03)	12.77 (2.65)	0.9105
	**Concentric hypertrophy**	7 (87.5%)	58 (82.9%)	0.581
	**LVOT obstruction**	0 (0%)	2 (2.9%)	0.877
	**Systolic impairment**	1 (12.5%)	5 (7.1%)	0.701
	**Diastolic impairment**	1 (12.5%)	5 (7.1%)	0.715
**Last clinical assessment (n=76)**	**NYHA >2**	1 (12.5%)	10 (10.3%)	0.867
	**Mean MLVWT (+/-SD)**	12.82 (1.73)	13.01 (3.10)	0.883
	**Concentric hypertrophy**	7 (87.5%)	51 (72.9%)	0.792
	**LVOT obstruction**	0 (0%)	1 (1.4%)	0.782
	**Systolic impairment**	3 (37.5%)	1 (1.43%)	<0.001
	**Diastolic impairment**	3 (37.5%)	7 (10.0%)	0.074

NYHA = New York Heart Association, LVOT = left ventricular outflow tract

References

1. Elliott PM, Anastasakis A, Borger MA, Borggrefe M, Cecchi F, Charron P, et al. 2014 ESC Guidelines on diagnosis and management of hypertrophic cardiomyopathy: the Task Force for the Diagnosis and Management of Hypertrophic Cardiomyopathy of the European Society of Cardiology (ESC). European heart journal. 2014;35(39):2733-79.
2. Nagueh SF, Smiseth OA, Appleton CP, Byrd BF, 3rd, Dokainish H, Edvardsen T, et al. Recommendations for the Evaluation of Left Ventricular Diastolic Function by Echocardiography: An Update from the American Society of Echocardiography and the European Association of Cardiovascular Imaging. Journal of the American Society of Echocardiography : official publication of the American Society of Echocardiography. 2016;29(4):277-314.
3. O'Leary PW, Durongpisitkul K, Cordes TM, Bailey KR, Hagler DJ, Tajik J, et al. Diastolic ventricular function in children: a Doppler echocardiographic study establishing normal values and predictors of increased ventricular end-diastolic pressure. Mayo Clinic proceedings. 1998;73(7):616-28.
4. Wyman Lai LM, Meryl Cohen, and Tal Geva Echocardiography in Pediatric and Congenital Heart Disease: From Fetus to Adult: Wiley Blackwell; 2009.
5. Rijnbeek PR, Witsenburg M, Schrama E, Hess J, Kors JA. New normal limits for the paediatric electrocardiogram. European heart journal. 2001;22(8):702-11.
6. Sokolow M, Lyon TP. The ventricular complex in left ventricular hypertrophy as obtained by unipolar precordial and limb leads. 1949. Annals of noninvasive electrocardiology : the official journal of the International Society for Holter and Noninvasive Electrocardiology, Inc. 2001;6(4):343-68.
7. Taran LM, Szilagyi N. The duration of the electrical systole, Q-T, in acute rheumatic carditis in children. Am Heart J. 1947;33(1):14-26.
