# Supplementary figures and images for: Friedreich’s ataxia-associated childhood hypertrophic cardiomyopathy: a national cohort study
Source: Arch Dis Child. 2021 Oct 5;107(5):450–5. doi: 10.1136/archdischild-2021-322455 (PMC9046745; doi:10.1136/archdischild-2021-322455)

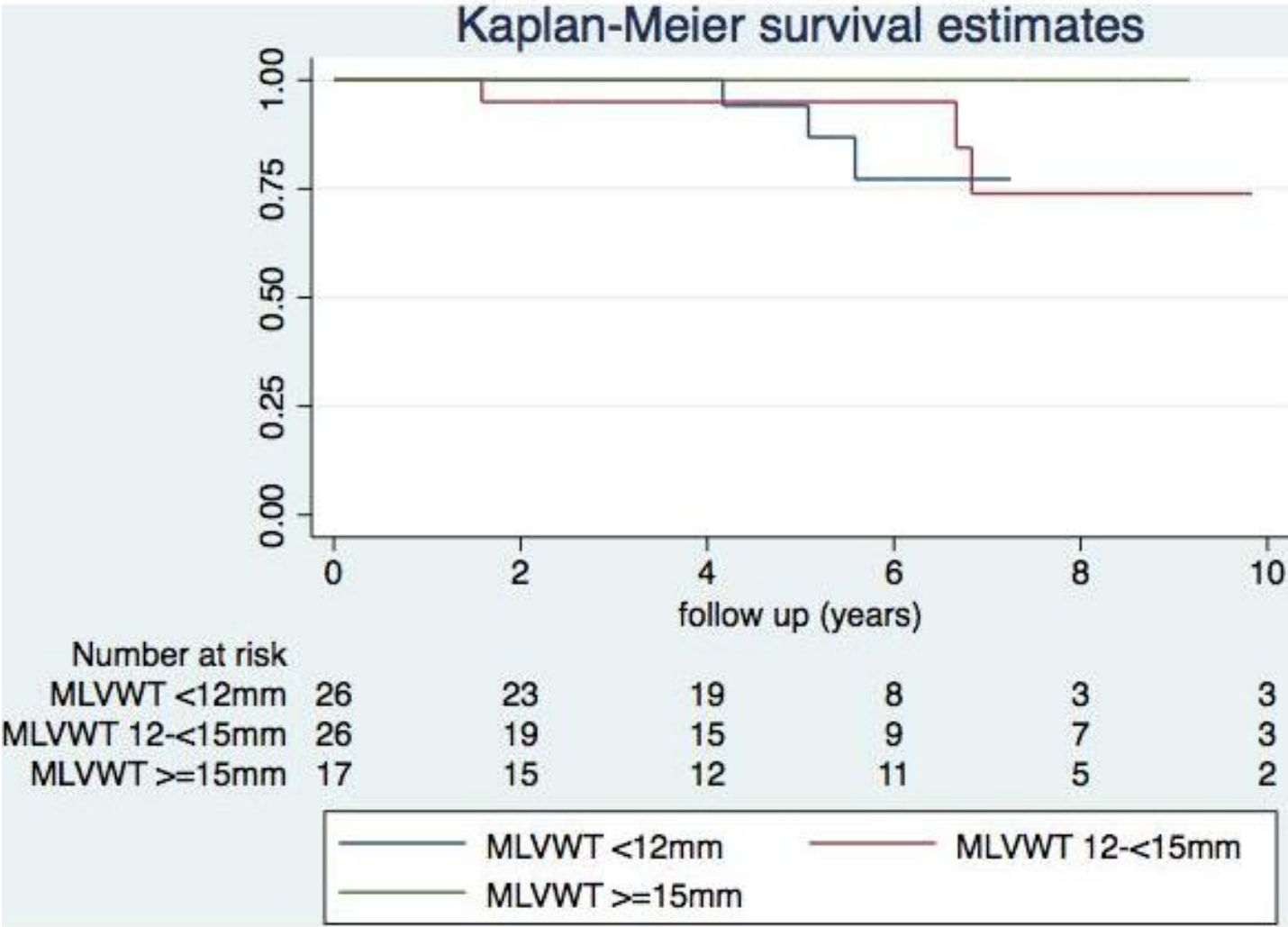

Supplement: Supplementary data [file archdischild-2021-322455supp002.pdf]
